# Special Issue “Chronic HCV Infection: Clinical Advances and Eradication Perspectives”

**DOI:** 10.3390/jcm11020359

**Published:** 2022-01-12

**Authors:** Maria Carla Liberto, Nadia Marascio

**Affiliations:** Unit of Microbiology, Department of Health Sciences, “Magna Graecia” University, 88100 Catanzaro, Italy

The latest report of global hepatitis estimated 58 million people with Hepatitis C virus (HCV) chronic disease and 1.5 million newly infected subjects per year [1]. In 2016, the World Health Organization (WHO) proposed a plan to reduce new infections and related deaths by 2030 [1]. However, the current severe acute respiratory syndrome coronavirus 2 (SARS-CoV-2) pandemic has determined a reallocation of public health resources, with a consequent delay in the hepatitis elimination program, already documented in Egypt and Italy [2]. In this Special Issue, we discuss the HCV eradication perspective related to the global situation before and during the ongoing pandemic. Direct-acting antiviral (DAA) agent efficacy, diagnostic methods and screening policy have all been evaluated via seven papers, six original articles and one review. 

The keywords with respect to the WHO plan are timely diagnosis and effective treatment for all infected individuals. The homeless and people who inject drugs (PWID), mono- or co-infected with HCV, have poor access to screening tests, medical care and showed a high reinfection rate after sustained viral response (SVR) [3]. In Italy, between January and June 2019, an observational study linked to these specific risk groups was carried out. The out-of-hospital model was able to guarantee better adherence to antiviral treatment and prevention of new HCV infections compared to the in-hospital model. Standard approaches need to be integrated with new healthcare strategies to achieve elimination of infection in the general, as well as in the neglected population [4].

Several studies: Using mathematical methods, an attempt was made to trace HCV elimination in different countries, highlighting tailored national interventions to achieve this goal [5]. Taking into account overall population, viremic patients, new diagnoses and other parameters to perform Model Base-Case, van Dijk and co-workers reported two main scenarios in the Netherlands. In the Status Quo scenario, the HCV target was set for 2027, while in the Gradual Decline scenario, for 2032. Interestingly, COVID-19 scenarios showed an increased number of decompensated cirrhosis and hepatocellular carcinoma (HCC) without significant delay in HCV eradication [6]. HCV infection is diagnosed by serological and molecular tests, while treatment and prognosis are related to liver damage and comorbidities [7,8]. Even if liver biopsy is the gold standard, conventional ultrasonography (US) and vibration-controlled transient elastography (VCTE) are noninvasive and cost-efficient methods currently adopted to measure fibrosis and steatosis progression. Florea et al. believed that performance of VCTE was superior to the conventional US technique due to the high negative predictive value and greater specificity. In the near future, VCTE could be very useful for risk prediction of HCC in HCV positive patients [9]. HCV is associated with hepatic and extra-hepatic illness, such as rheumatic diseases, which can be alleviated after antiviral therapy [8,10]. Cheng and coauthors, conducting a nationwide population study, reported how interferon (IFN) therapy did not mitigate rheumatic disease risk. On the contrary, the IFN-free treatment effect after SVR needs to be further investigated [10].

Pan-genotypic therapy is used to treat HCV infected people independently of the genotype resistance test [11]. Nevertheless, real life data show that DAA efficacy can be influenced by resistance-associated substitutions (RASs) carried by target genomic regions. Between 2015 and 2016, we enrolled 41 HCV1b positive patients who reported surgical intervention, unsafe use of glass syringes, and dental treatment as risk factors. We analyzed the HCV1b viral isolates to evaluate the presence of RASs in NS5A and NS5B amplicons. In particular, in 36.5% of NS5B sequences, L159F was carried alone and in 19.5% was found in combination with C316N, both associated with lower response to sofosbuvir (SOF). On the other hand, three NS5A sequences displayed the Y93H RAS currently responsible for many DAA regimen failures [12]. In 2017, the ledipasvir (LDV)/SOF combination was approved by the European Medical Agency (EMA) and the Food and Drug Administration (FDA) to cure children 12–17 years old. Pokorska-Śpiewak et al. reported efficacy and safety of LDV/SOF therapy in adolescents with HCV chronic diseases infected by HCV1 or HCV4. The study had limitations on data collection due to the SARS-CoV-2 pandemic [13]. These results are in line with our previously published paper. Two HCV4 pediatric patients achieved SVR, although viral isolates carried both the L28M and M31L NS5A RASs [14]. Despite the high SVR rate in pan-genotypic regimens, at present HCV3 is the most difficult-to-treat genotype, especially in cirrhotic and DAA-treated patients. However, real-world data reported by Zarębska-Michaluk and co-authors showed the higher effectiveness of glecaprevir/pibrentasvir (96%) compared to SOF/velpatasvir (VEL) (93%) and to SOF/VEL + ribavirin (79%) regimens [15].
